# How silicene on Ag(111) oxidizes: microscopic mechanism of the reaction of O_2_ with silicene

**DOI:** 10.1038/srep17570

**Published:** 2015-12-03

**Authors:** Tetsuya Morishita, Michelle J.S. Spencer

**Affiliations:** 1 Nanomaterials Research Institute, National Institute of Advanced Industrial Science and Technology (AIST), Central 2, 1-1-1 Umezono, Tsukuba, Ibaraki 305-8568, Japan; 2 School of Applied Sciences, RMIT University, GPO Box 2476, Melbourne, Victoria 3001, Australia

## Abstract

We demonstrate, using first-principles molecular-dynamics simulations, that oxidation of silicene can easily take place either at low or high oxygen doses, which importantly helps clarify previous inconsistent reports on the oxidation of silicene on the Ag(111) substrate. We show that, while the energy barrier for an O_2_ molecule reacting with a Si atom strongly depends on the position and orientation of the molecule, the O_2_ molecule immediately dissociates and forms an Si-O-Si configuration once it finds a barrier-less chemisorption pathway around an outer Si atom of the silicene overlayer. A synergistic effect between the molecular dissociation and subsequent structural rearrangements is found to accelerate the oxidation process at a high oxygen dose. This effect also enhances self-organized formation of *sp*^3^-like tetrahedral configurations (consisting of Si and O atoms), which results in collapse of the two-dimensional silicene structure and its exfoliation from the substrate. We also find that the electronic properties of the silicene can be significantly altered by oxidation. The present findings suggest that low flux and low temperature of the oxygen gas are key to controlling oxidation of silicene.

The two-dimensional (2D) structure of silicon (Si), which is a graphene analog, is called “silicene”. It has attracted intense interest since its successful synthesis in the laboratory on a variety of substrates^1^,^2^,^3^,^4^,^5^,^6^,^7^,^8^,^9^ and it has been argued that silicene could have unique electronic properties such as massless Dirac Fermion behavior, as graphene does^10^. Silicene is thus expected to possess great potential for future applications such as nanoscale electronic devices. In contrast to graphene, silicene is known to take a variety of 2D configurations on a substrate (such as Ag or Al), including those with a periodicity of 4 × 4, 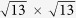
11, and even a configuration consisting of 3-, 4-, 5-, and 6-sided polygons^8^. This variety of structures gives silicene more flexible properties than graphene.

Silicene also has the great advantage of easy integration into existing circuitry that is already based on Si technology. The application of silicene to nanoscale devices is, however, currently hindered by the existence of unsaturated (dangling) bonds on its surface, which makes it highly reactive under atmospheric conditions. Because of this, pristine silicene can only be grown under vacuum on a substrate; silicene is only found to be free from contamination or oxidation in air when capped by e.g., organomolecules^12^,^13^,^14^,^15^,^16^. It is, however, crucial to be able to control the stability of silicene under various conditions in order to fabricate silicene-based nanodevices. Elucidating the stability of silicene under atmospheric conditions, and especially its resistance toward oxidation, is thus urgently required.

Only a few theoretical studies have examined oxidation of silicene, however, these have focused on free-standing silicene, and not silicene on a substrate^17^,^18^,^19^,^20^. These studies, including the work by Wang *et al.*^17^, Ozcelik *et al.*^18^ and Liu *et al.*^19^,^20^, reported that free-standing silicene is unstable in O_2_ as O_2_ will readily dissociate on silicene, in a barrier-less process, to form stable Si-O bonds. The resulting silicene oxides may retain the honeycomb lattice of silicene or distort it.

For silicene on a substrate, in contrast, previous experimental studies have reported contradictory observations on the oxidation of silicene exposed to oxygen gas (O_2_). De Padova *et al.*^21^ and Molle *et al.*^22^ claimed that silicene (in the form of a nanoribbon and nanosheet, respectively) possesses low reactivity towards O_2_ up to 1000 Langmuir (L). In contrast, more recent experiments by Du *et al.*^23^,^24^ demonstrated that silicene on Ag(111) exhibits a high reactivity towards oxygen, observing incorporation of oxygen atoms into the silicene honeycomb network even at low gas coverages (e.g. 10–20 L). Furthermore, they obtained highly oxidized silicene under an oxygen exposure of 60 L, and even amorphous-like silicene oxides at 600 L, at which crumpling of the silicene overlayer was observed, suggesting its exfoliation from the underlying substrate^23^.

This glaring contradiction highlights the need to illuminate the surface morphology and dynamical processes occurring at an atomistic level in the early stage of silicene oxidation. Such detail, however, is extremely difficult to achieve in experiment. What is needed, therefore, is a computational approach that can unveil the dynamical changes of the atomic configuration during the oxidation process. In our previous work with Xu *et al.*^24^ we performed DFT calculations of oxidized silicene on the Ag(111) surface, that represented the structure determined from the STM experiments, and its stability at a simulation temperature of 300 K. We did not, however, model the oxidation process itself, but instead started with the oxidized overlayer.

Here, we report a first-principles molecular-dynamics (FPMD) study of oxidation of 4 × 4 silicene on Ag(111) with low (~0.1 monolayer (ML)) and high (~0.44 ML) oxygen coverages. To the best of our knowledge, this is the first attempt to reveal the dynamical process of oxidation of the silicene overlayer on the Ag(111) substrate. We show that an O_2_ molecule can easily react with the Si atoms in the silicene overlayer on Ag(111). In particular, an O_2_ molecule is able to find barrier-less pathways to oxidation when it is within a distance of ~3 Å from a substrate-side Si atom (i.e. a Si atom that is located closest to a substrate atom). Sequential displacements or movements of specific atoms, which we call a “chain-like reaction”, are found to play a significant role in the oxidation process at high oxygen coverages. Also shown in our work, is that the electronic properties of silicene can be significantly altered by oxidation, which gives us some hints for the potential applications of silicene oxides. We note that there are two types of 4 × 4 superstructures of silicene on the Ag(111) surface, namely 4 × 4-*α*^3^,^25^ and 4 × 4-*β*^25^. Since oxidation of the former silicene overlayer was examined in most of the previous experiments^22^,^23^,^24^, we use the same 4 × 4 structure for silicene on Ag(111) in our simulations reported here.

## Results

### Oxidation at a low oxygen dose

**Figure 1:**
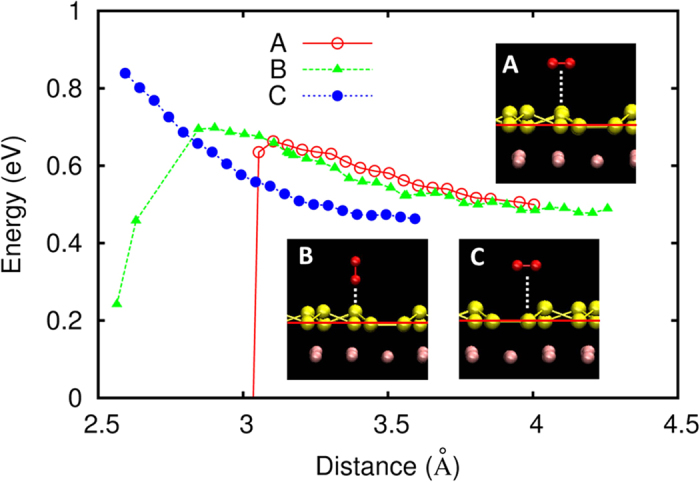
Energy as a function of the O_2_-silicene distance for Case (A), Case (B), and Case (C) (see text). The initial configuration for each case is shown in the insets. The red, yellow, and pink atoms indicate oxygen, silicon, and silver atoms, respectively.

A single O_2_ molecule was introduced into the system of 18 Si and 80 Ag atoms to model the reaction of oxygen with the 4 × 4 silicene overlayer at a low oxygen coverage. This corresponds to an O_2_ coverage of 0.11 ML. Firstly, we constructed the energy profile of the O_2_/silicene/Ag(111) system as a function of the distance between the silicene overlayer and the O_2_ molecule. The following three arrangements are considered (Fig. 1): (A) an O_2_ molecule approaching an outer Si atom of the silicene overlayer, keeping the molecular axis parallel to the silicene surface, (B) an O_2_ molecule approaching an outer Si atom, keeping the molecular axis perpendicular to the surface, and (C) an O_2_ molecule approaching an Ag-side Si atom, keeping the molecular axis parallel to the surface. At each distance, the positions of the O atoms are allowed to relax (while keeping the molecular orientation fixed), in addition to relaxing the positions of the Si and Ag atoms. The distance between the silicene overlayer and the O_2_ molecule was measured from the Ag-side Si atoms (denoted by the red lines in the insets of Fig. 1. In Case B, the distance is measured from the Ag-side Si atom to the Ag-side O atom).

The energy profile for Case A and Case B in Fig. 1 exhibits a monotonic increase in energy as the O_2_ molecule approaches the silicene overlayer while keeping its molecular orientation. The energy barriers are estimated to be 0.16 eV for Case A at a distance of 3.1 Å, and 0.20 eV for Case B at a distance of 2.9 Å. The sharp drop in energy seen at shorter distances is attributed to the formation of Si-O bonds, where the outer Si atom is lifted upward to form the Si-O bonds.

In contrast to Case A and Case B, such an energy drop is not seen for Case C, wherein an Si-O bond is not formed. In fact, the O_2_ molecule moved horizontally as if it experienced a repulsive force from the Ag-side Si atom when the distance was decreased to ~2.6 Å. This indicates that the reactivity with oxygen is quite different between the outer- and Ag- side Si atoms, which would come from the fact that the dangling bond on the latter is effectively removed by the Ag atoms while that on the former is left intact.

**Figure 2:**
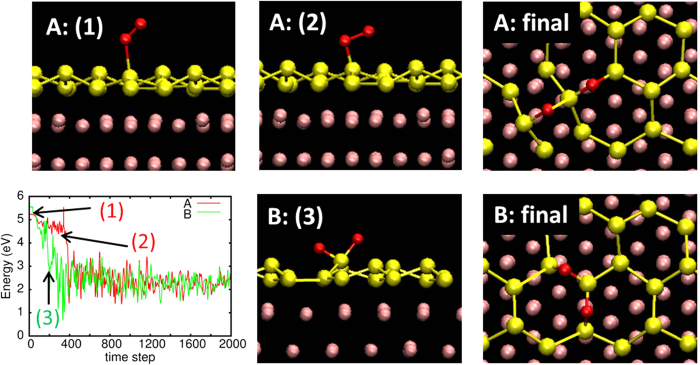
Snapshots from the FPMD runs for Case (**A**) and Case (**B**) (see text). The corresponding time evolution of the energy is shown in the lower-left panel in red (Case **A**) and green (Case **B**) (note that the time step is 1 fs). The red, yellow, and pink atoms indicate oxygen, silicon, and silver atoms, respectively.

It is clear from Fig. 1 that there exists an energy barrier when an O_2_ molecule approaches the silicene overlayer while keeping its orientation vertical or parallel to the surface. The barrier, however, may be reduced by approaching the surface in different orientations. We thus performed FPMD simulations (under the NVE condition) to see if the O_2_ molecule itself was able to find other pathways with lower (or no) barriers to react with the Si atoms. Figure 2 shows the snapshots from the FPMD simulations runs that were performed for Case A and Case B, along with the time evolution of the energy from the same FPMD runs. The simulations were started with the O_2_ molecule located at a distance of 3.26 Å (for Case A) or 3.10 Å (for Case B). We found that, even with a zero initial velocity, the O_2_ molecule reacted with the Si atoms to form an Si-O-Si configuration in both cases.

At the beginning of the reaction, the O_2_ molecule started tilting towards the surface and then bonded to a Si atom forming surface bound Si-O_2_, in both cases as shown in Panel A:(1) [the snapshot is taken at the time denoted by “(1)” in the lower-left panel (the red line)]. The Si-O_2_ configuration where the O_2_ bond is at an angle to the surface [Panel A:(1) and A:(2)] was maintained for a while in Case A (see the red line in the lower-left panel), but the O_2_ molecule finally found a pathway to react with the Si atom and dissociate, resulting in the configuration given in “Panel A: final”.

In Case B, the O_2_ molecule dissociated soon after the tilting occurred [see Panel B:(3); snapshot taken at the time denoted “(3)” in the lower-left panel (the green line)], and each O atom finally adsorbs in a bridge site creating an Si-O-Si surface bound structure (Panel B: final) at the end of the FPMD run. Hence, the O_2_ molecule was again able to find a pathway to the dissociation reaction.

The Si-O bond length in both the Case A and Case B final configurations ranged from 1.63 to 1.76 Å, which is slightly longer than the Si-O bond length in crystalline SiO_2_ (1.62 Å), but is in good agreement with the previous theoretical results of free-standing silicene^17^,^18^,^19^,^20^. The slightly longer Si-O bond length in the oxidized silicene is due to the high buckling of the honeycomb lattice incorporating the O atoms. The energy significantly decreases in both reaction processes, in which an energy equivalent to ~270 K is released indicating this is an exothermic process.

Our FPMD simulations, therefore, clearly show that there are low-energy barrier pathways for the reaction of O_2_ with silicene. The formation of a configuration where an outer Si atom has a tilted O_2_ molecule adsorbed on it is the key to finding a pathway to the oxidation reaction. The formation of such a configuration could be rationalized as follows; electron donation from the Si atoms takes place when an O_2_ molecule dissociates, which stabilizes the Si-O bond. (This is consistent with a previous calculation of the oxidation of Si(001)^26^, and will also be demonstrated in our calculations later.) As an O_2_ molecule is considered to have a double bond, such a transfer of electrons may easily occur when an O atom of the molecular oxygen adsorbs close to an outer Si atom. Therefore, the surface bound Si-O_2_ configuration as in Fig. 2A:(1) is favored in the oxidation process. This, in turn, makes the hollow site of the Si_6_ ring less favorable to the O_2_ molecule. Note that the center of the O_2_ molecule, on the other hand, does not favor the outer Si atom, because it is unlikely that electron transfer will take place between them due to electron repulsion. This is consistent with the fact that there exists an energy barrier when the center of an O_2_ molecule approaches the outer Si atom orientated parallel to the surface, as in Case A (see Fig. 1). Also note that a barrier-less reaction leading to the formation of the Si-O_2_ configuration has also been observed in a previous DFT calculation of free-standing silicene^20^, suggesting that the Ag(111) substrate does not greatly affect the reactivity of the silicene overlayer toward an O_2_ molecule.

We also performed a FPMD run for Case C with the O_2_ molecule initially located at a distance of 2.64 Å (see Fig. 1) from the silicene. The O_2_ molecule immediately started moving away from the silicene overlayer even with a zero initial velocity. This markedly contrasts with Case A and Case B. We repeated the FPMD simulation with a slightly different initial position of the O_2_ molecule (where it started at a distance of 3.1 Å and a different orientation of the molecular axis, while still keeping it parallel to the silicene overlayer). The molecule again moved away from the surface without adsorbing or reacting with the silicene. It thus appears that there is not a barrier-less pathway to oxidation around the Ag-side Si atoms, or the pathway, if any, is very narrow for O_2_ to enter without a guiding force. This finding is consistent with the recent experimental observation that oxidation always starts with the outer Si (top-layer Si) atoms^23^.

The different behavior observed in Case A, B and C indicates that the energy landscape felt by the O_2_ molecule above the silicene overlayer is highly rugged and depends on the surface morphology, so that exploring the landscape is necessary for O_2_ to react with the silicene. This means that the O_2_ molecule should have a certain amount of kinetic energy to be able to explore the landscape. Oxidation may thus be possibly suppressed if the temperature can be kept very low. We note that such a dependence of chemical reactivity on the surface morphological details is also observed in the oxidation process of the Si(001) surface^26^.

While the resultant configurations obtained in Case A and Case B are slightly different, the Si-O-Si configuration is formed in both cases, and is consistent with the previous experimental result^23^. The energy difference between these configurations at 0 K is only ~0.17 eV/system (i.e. ~17 K); the “B configuration” has lower energy. Because of this small difference, either configuration could be formed in experiment at low oxygen coverages. Note that we confirmed that formation of the Si-O-Si configuration is not accidental by repeating the FPMD simulations multiple times for Case A and Case B, as well as for Case C. This suggests that the reactions we present here are not a statistical anomaly.

**Figure 3:**
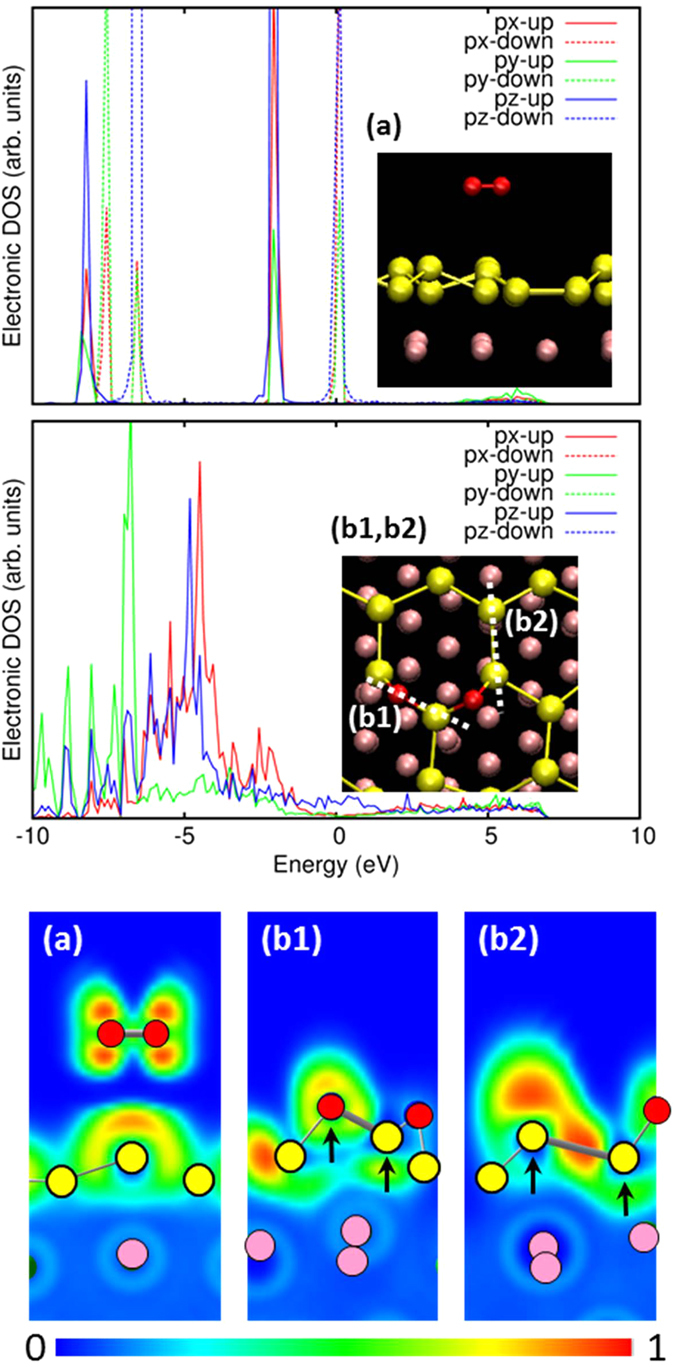
(Upper panel) Density of state (DOS) for the *p* electrons of the O_2_ molecule indicated in the structure (inset). (Middle panel) DOS for the *p* electrons of the dissociated oxygen atoms shown in the structure (inset). The zero of energy is set at the Fermi energy. (Lower panel) ELF plots for Slices (**a**,**b1**,**b2**) indicated in the insets in the upper and middle panels. Slice (**a**) cuts through the O_2_ bond axis, while Slices (**b1**,**b2**) cut through the Si-O and Si-Si bonds, respectively, as indicated by the arrows and by the dashed lines in the inset of the middle panel. The red, yellow, and pink atoms indicate oxygen, silicon, and silver atoms, respectively.

The electronic properties of the O_2_ molecule before and after its reaction with the silicene (corresponding to the B:final structure) are presented in Fig. 3. The upper panel of Fig. 3 shows the density of state (DOS) for the *p* electrons of the O_2_ molecule before the reaction, in the configuration displayed in the inset panel. The molecule is clearly spin-polarized, as is expected for the ground state of an O_2_ molecule (triplet state). In contrast, the DOS for the dissociated O atom, corresponding to the O-Si-O adsorbed configuration (inset panel of the lower DOS plot) shows a spin-unpolarized characteristic; the up-spin and down-spin bands are fully degenerate due to the formation of the Si-O-Si configuration.

The lower panel of Fig. 3 shows a plot of the electron localization function (ELF)^27^ that gives an indication of the probability of electron localization as measured between 0 and 1, with 1 showing a high probability of covalent bonding. The subpanels (a), (b1), and (b2) display the color plot of the ELF for the slices indicated in the insets of the DOS panels of Fig. 3 [Slice (a) includes the whole O_2_ molecule, while Slices (b1) and (b2) include the Si-O and Si-Si bonds, respectively, with the O and Si atoms that the slice passes through denoted by the arrows]. The notable point is that the electron localization in the Si-Si bond (b2) remains high, while that in the Si-O bond (b1) is lower. This indicates that the Si-O bond is rather ionic compared to the Si-Si bond. In fact, our Bader charge analysis shows that each of the O atoms gains electrons of ~1.5|*e*| from its adjacent Si atoms after forming the Si-O-Si configuration. Thus the electronic property associated with the intermediate *sp*^2^/*sp*^3^ bonding in the silicene overlayer is significantly altered by the oxidation.

### Oxidation at a high oxygen dose

Our FPMD calculations show that sequential reactions involving more than one O_2_ molecule are the key to understanding the oxidation process of silicene at high oxygen coverages. Specifically, the oxidation process is dominated by a “chain-like reaction” involving multiple O_2_ molecules, which will be detailed below.

In the FPMD simulation performed at a high oxygen coverage, 16 O_2_ molecules are introduced into the system of 72 Si and 320 Ag atoms, corresponding to an oxygen coverage of 0.44 ML on Si/Ag(111). This coverage is consistent with our previous preliminary calculations^24^ that corresponded to a coverage of 0.5 ML, and is sufficient to follow the dynamical changes of the atomic configuration in the early stage of the oxidation. All the O_2_ molecules were initially evenly positioned across the lateral directions of the surface, above the silicene overlayer (See Fig. S1 of the Supporting Information). The distances between the O_2_ molecules and the silicene overlayer were in the range of 4.42–6.43 Å. The initial velocities at ~300 K were assigned to each O_2_ molecule, and were adjusted to move the O_2_ molecules toward the silicene overlayer. The 4 × 4 structure,^3^ without any defects, was employed as the initial structure for the silicene overlayer on the Ag(111) surface. The initial velocities of the Si and Ag atoms were also set to have a kinetic energy of 300 K, and the temperature of the whole system was then kept at 300 K throughout the FPMD run. We note that the effective oxygen flux in the simulation would be extremely high compared to experiment because of the short simulation time (3.5 ps).

**Figure 4:**
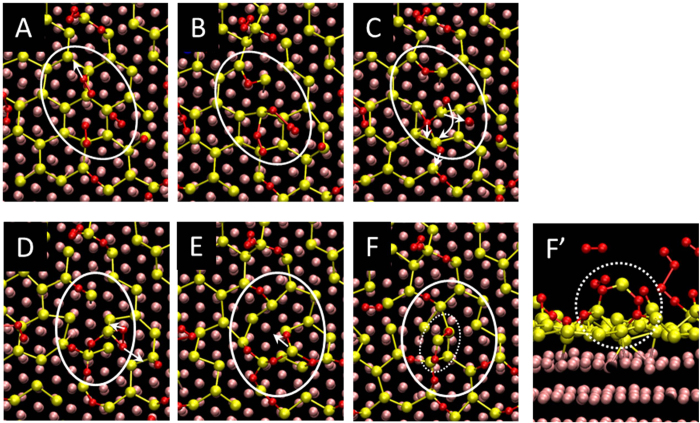
A typical sequence of “chain-like reaction” on the silicene overlayer involving multiple O_2_ molecules (top views). The process proceeds from Panel A to Panel F: (**A,B**) an O_2_ molecule dissociates and Si-O configurations are created; (**C,D**) structural rearrangements with substantial atomic displacements take place; (**E,F**) a new reaction site with a 3D-like configuration is created. The white arrows indicate where an oxygen atom will move to in the next step of the reaction. The white circle in each panel focuses the process described above, and each circle contains the same oxygen atoms throughout the sequence (**A–F**). Panel F′ displays the side view of Panel F; the white dashed circles in Panel F and F′ show the new reaction site created during this sequence. It took 0.82 ps to proceed from (**A–F**). The red, yellow, and pink atoms indicate oxygen, silicon, and silver atoms, respectively. (The panels displaying the same atomic configurations viewed from a different angle are given in Fig. S2, Supporting Information.)

A typical “chain-like reaction” process is displayed in Fig. 4, wherein a series of reactions proceeds from A through to F. The white circles in the panels of Fig. 4 denote the O_2_ reactions or structural rearrangements focused on in each panel. The same oxygen atoms are included in the white circles throughout A to F.

We found, that by looking at the process in detail, the following three steps play an important role in the oxidation at high O_2_ coverages: (1) O_2_ molecules dissociate on the silicene surface and Si-O bonds are created, some of them acting as new reaction sites [A, B], (2) structural rearrangements with substantial atomic displacements follow the O_2_ reactions [C, D], (3) new reaction sites are created during the structural rearrangements [E, F] (for example, an under-coordinated Si atom is lifted up toward the vacuum space in the substantial structural rearrangement as in Fig. 4(F′), which is a highly reactive site towards O_2_ molecules).

When an O_2_ molecule approaches an outer Si atom, the O_2_ molecule immediately dissociates forming Si-O bonds; this step is followed by structural rearrangements that create new reaction sites. This series of actions enhances the subsequent O_2_ reactions, since available reaction sites are repeatedly formed, resulting in a “chain-like” oxidation process. (In fact, we found that a “chain-like reaction” similar to Fig. 4 took place simultaneously on a different part of the silicene overlayer. See Fig. S3.) This reaction leads to collapse of the original honeycomb 2D structure and formation of 3D-like bonded configurations [e.g. see Fig. 4(F′)]. Since highly oxidized silicene showed a tendency towards exfoliation from the Ag substrate in the previous experiment^23^,^24^, we suggest that the growth of such 3D-like configurations may be responsible for the exfoliation.

**Figure 5:**
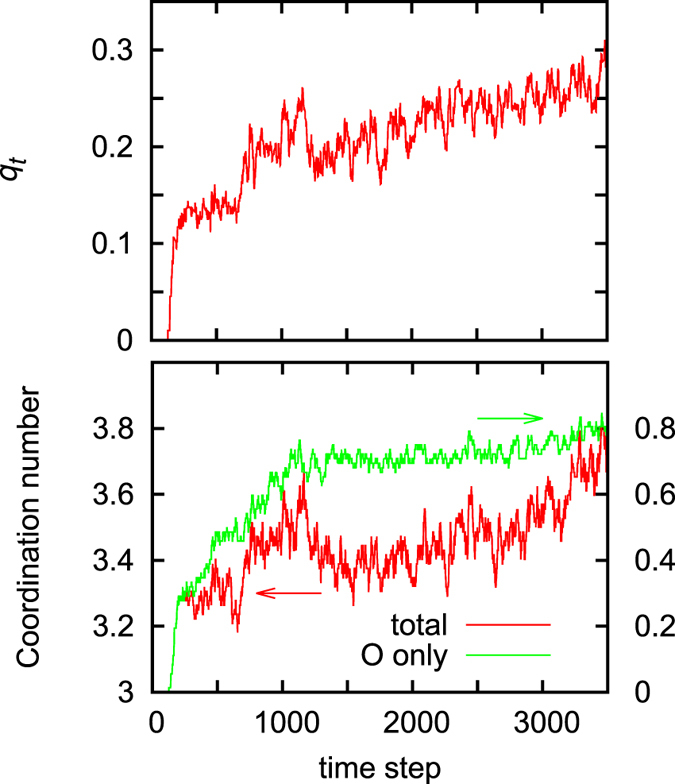
(Upper panel) Time evolution of the order parameter *q*_*t*_ (that estimates the tetrahedrality) during the oxidation process. (Lower panel) Time evolution of the coordination number, *N*_*c*_, of Si: *N*_*c*_ counting both Si and O atoms (red line) and *N*_*c*_ counting O atoms only (green line).The time steps is 1 fs.

It is not surprising that the 3D-like structural arrangements are formed during the oxidation process, considering that an O-Si-O configuration favors the tetrahedral bonding that is seen in the SiO_2_ crystal. Figure 5 shows the tetrahedrality of the structural configuration composed of a Si atom with its four neighboring atoms (either Si or O). Here, the order parameter *q*_*t*_ is calculated to estimate the tetrahedrality^28^,^29^, *q*^*k*^_*t*_  is defined for each Si atom (*k*th atom) at each MD step as 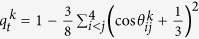, where *θ*^*k*^_*ij*_ is the angle between the vectors that join a central Si atom with its *i*th and *j*th nearest neighbors (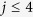). *q*_*t*_ is then obtained by averaging *q*^*k*^_*t*_  over all the Si atoms as 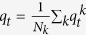, where *N*_*k*_ is the number of the Si atoms.

The upper panel of Fig. 5 shows the time evolution of *q*_*t*_ during the oxidation process. *q*_*t*_ takes a value of 0 (by definition) before the O_2_ reactions start since all the Si atoms have three neighboring atoms only. Once the reactions start (at a time step of ~200), however, O atoms become incorporated into the Si honeycomb network, forming Si-O bonds and inducing the structural rearrangements. This results in the sharp increase and subsequent steady growth of *q*_*t*_, indicating that the number of tetrahedral configurations monotonically increases. That is, the degree of *sp*^2^ character of the bonding in the silicene 2D structure is gradually reduced, and a tetrahedral 3D structure having an enhanced *sp*^3^ character grows instead.

Interestingly, the growth of *q*_*t*_ does not show a direct correlation with that of the coordination number of Si (*N*_*c*_). The lower panel of Fig. 5 shows the time evolution of *N*_*c*_ [counting both Si and O atoms (red lines), or O atoms only (green lines) as the neighboring atoms]. Similarly to *q*_*t*_, *N*_*c*_ sharply increases and then grows steadily. The monotonic growth, however, ceases at a time step of ~1100, followed by only a slight increase. The number of O atoms counted as the neighboring atoms (green line) is rather constant after the growth stage. This reflects the fact that most of the O_2_ molecules in the system had already reacted with the silicene overlayer and had been incorporated into the Si-Si bond network at the growth stage. As can be seen in the upper panel of Fig. 5, however, *q*_*t*_ continues increasing even after the growth stage (at a time step > 1100). It is thus considered that the structural rearrangement recovering the tetrahedrality can proceed without the supply of O_2_ molecules, once it is triggered by oxidation. This implies that a high oxygen flux within a short timeframe would be sufficient to enhance the formation of the 3D oxidized configurations and thus exfoliation of the silicene overlayer. The total *N*_*c*_ (counting both Si and O atoms as the neighboring atoms) shows even a slight decrease at time step 1100–1500. This indicates that under-coordinated Si atoms are newly generated in the structural rearrangements, as has been discussed before [e.g. Fig. 4(F′)]. The total *N*_*c*_ then exhibits a slight increase again after a time step of ~1500, induced by further rearrangements or capture of O_2_ molecules that were still intact.

It is worth noting that, because of this high oxygen dose, some of the O_2_ molecules could form dimers or trimers via direct intermolecular interactions before reacting with the silicene overlayer. Such O_2_ aggregates facilitate the occurrence of the chain-like reaction. This indicates that uniformly oxidized silicene may not be easily obtained at a high oxygen coverage, but instead would result in exfoliation and emergence of the bare Ag surface. A low flux, with a long time exposure to oxygen may thus be needed to form uniformly oxidized silicene and hence formation of a silicene oxide sheet.

**Figure 6:**
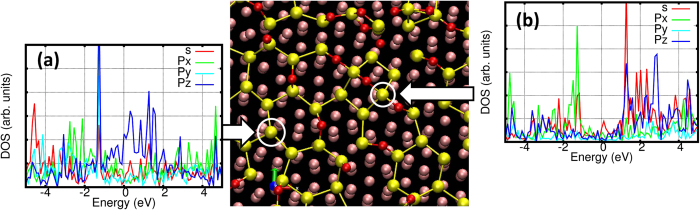
Decomposed DOS for (**a**) a threefold-coordinated Si and (**b**) a fourfold-coordinated Si in the oxidized silicene (as indicated by the bold arrows and white circles). The zero of energy in the DOS plots is aligned to the Fermi energy. The red, yellow, and pink atoms indicate oxygen, silicon, and silver atoms, respectively.

The atom-resolved DOS for the oxidized Si atoms at the high O_2_ coverage (Fig. 6) clearly show the change in electronic properties induced by the oxidation. Figure 6(a) shows the atom-resolved DOS for a Si atom having three neighboring Si atoms, as in the silicene honeycomb lattice. It is clear that the electronic bands near the Fermi energy have a high intensity and are dominated by the *p*_*z*_ electrons from the dangling bond on the Si atom, and show a metallic nature. In contrast, the atom-resolved DOS for a four-coordinated Si atom (with two O atoms and two Si atoms) having a highly tetrahedral configuration [Fig. 6(b)] shows completely different characteristics with the electronic bands near the Fermi energy being substantially reduced, especially those from the *p*_*z*_ electrons, due to capping the dangling bond with O atoms. We thus conclude that the metallic nature of silicene is reduced as oxidation proceeds. This tendency has also been observed in the recent experimental study^23^, which reports that a semiconducting nature for silicene could be realized by the oxidation process.

## Discussion

The present FPMD calculations reveal that there exist barrier-less oxygen chemisorption pathways around the outer Si atoms of the silicene overlayer. Though the pathways are not significantly wide for the O_2_ molecule, oxygen can easily react with a Si atom to form an Si-O-Si configuration, once the molecule finds an entrance to the pathway on the rugged energy landscape provided by the silicene overlayer. The resultant Si-O bond is ionic, rather than covalent, because of the charge transfer from the Si atom to the O atom. As a result, the nature of the intermediate *sp*^2^/*sp*^3^ bonding is substantially degraded, and a tetrahedral 3D configuration as seen in SiO_2_ crystals locally forms as the oxidation proceeds.

In the oxidation process involving multiple O_2_ molecules (representing a high oxygen dose), a synergistic effect between the molecular dissociation and the subsequent structural rearrangement is the key to understanding the atomistic mechanism of the oxidation process. A notable point is that the structural transformations resulting in highly tetrahedral configurations composed of Si and O atoms can proceed without a supply of O_2_ molecules, once the silicene overlayer is covered by oxygen of >~0.5 ML. This self-organized rearrangement should be one of the driving forces to accelerate exfoliation of the silicene overlayer from the Ag substrate at a high oxygen dose. Careful control of the oxygen flux is thus necessary to produce an oxidized silicene sheet maintaining its 2D morphology. Also, suppression of the oxidation process might be possible by maintaining the oxygen gas temperature at a low value.

Significantly, our results give some hints to help explain why the differences seen experimentally for the oxidation of silicene on the Ag(111) surface arose. We suggest from our work that a number of factors, such as oxygen coverage or dose, as well as reaction temperature, may alter the degree of oxidation of silicene. In particular, our results indicate that a different flux (or pressure) of oxygen gas could induce different oxidation processes. As the unit of Langmuir is the pressure of the gas times the time of exposure, different pressure conditions may lead to the same value in L. This means that experiments reporting the reaction of O_2_ with the silicene at exposures with the same Langmuir value may actually be using different pressure conditions. It is therefore highly possible that oxidation could proceed differently in the experiments given the same exposure.

Further, since the electronic properties can be altered by oxidation, as demonstrated in this work, control of the process is highly desirable so as to obtain non-oxidized, partially-oxidized, or fully-oxidized silicene. The present results are thus of great help to realize such control and to extend the potential range for the use of silicene in nanoscale devices under a variety of conditions, including metal/oxide semiconductor devices.

## Methods

The present FPMD calculations were performed within the framework of density functional theory as implemented in the Vienna Ab Initio Simulation Package (VASP)^30^. The exchange-correlation functional in the Perdew-Burke-Ernzerhof form^31^ was used and the ion-electron interaction was described by the projector augmented wave method^32^. Two systems were modeled based on the unit structure of 4 × 4 silicene on the Ag(111) surface (3 × 3 honeycomb silicene lattice on the 4 × 4 Ag(111) surface) with dimensions of 11.6496 Å × 11.6496 Å × 30.0 Å. One system is for a low oxygen coverage (0.11 ML) composed of the 1 × 1 unit structure (consisting of 2 O, 18 Si, and 80 Ag atoms), while the other system represents a high oxygen coverage (0.44 ML) composed of a 2 × 2 unit structure (consisting of 32 O, 72 Si, and 320 Ag atoms). The Ag substrate consists of five atomic layers with the bottom layer fixed. This model has been validated previously^8^,^24^. A plane-wave basis set with an energy cutoff of 400 eV was used with the following k-point mesh; 11 × 11 × 1 for the DOS calculations and 2 × 2 × 1 for the MD calculations of the 0.11 ML system, while a 2 × 2 × 1 mesh was used for the DOS calculations and Γ-point only for the MD calculations of the 0.44 ML system. A time step of 1 fs was used in the MD calculations for both systems.

## Additional Information

**How to cite this article**: Morishita, T. and Spencer, M.J.S. How silicene on Ag(111) oxidizes: microscopic mechanism of the reaction of O_2_ with silicene. *Sci. Rep.* **5**, 17570; doi: 10.1038/srep17570 (2015).

## Supplementary Material

Supplementary Information
