# Supplementary material for: How silicene on Ag(111) oxidizes: microscopic mechanism of the reaction of O2 with silicene
Source: Sci Rep. 2015 Dec 3;5:17570. doi: 10.1038/srep17570 (PMC4668378; doi:10.1038/srep17570)
Supplement: Supplementary Information [file srep17570-s1.pdf]

Supplementaty Information for

How silicene on Ag(111) oxidizes: microscopic mechanism of the reaction of O_2 with silicene

Tetsuya Morishtia[1] and Michelle J. S. Spencer[2]

[1] Nanomaterials Research Institute, National Institute of Advanced Industrial Science and Technology (AIST),
Central 2, 1-1-1 Umezono, Tsukuba, Ibaraki 305-8568, Japan
t-morishita@aist.go.jp

[2] School of Applied Sciences, RMIT University, GPO Box 2476,
Melbourne, Victoria 3001, Australia
michelle.spencer@rmit.edu.au

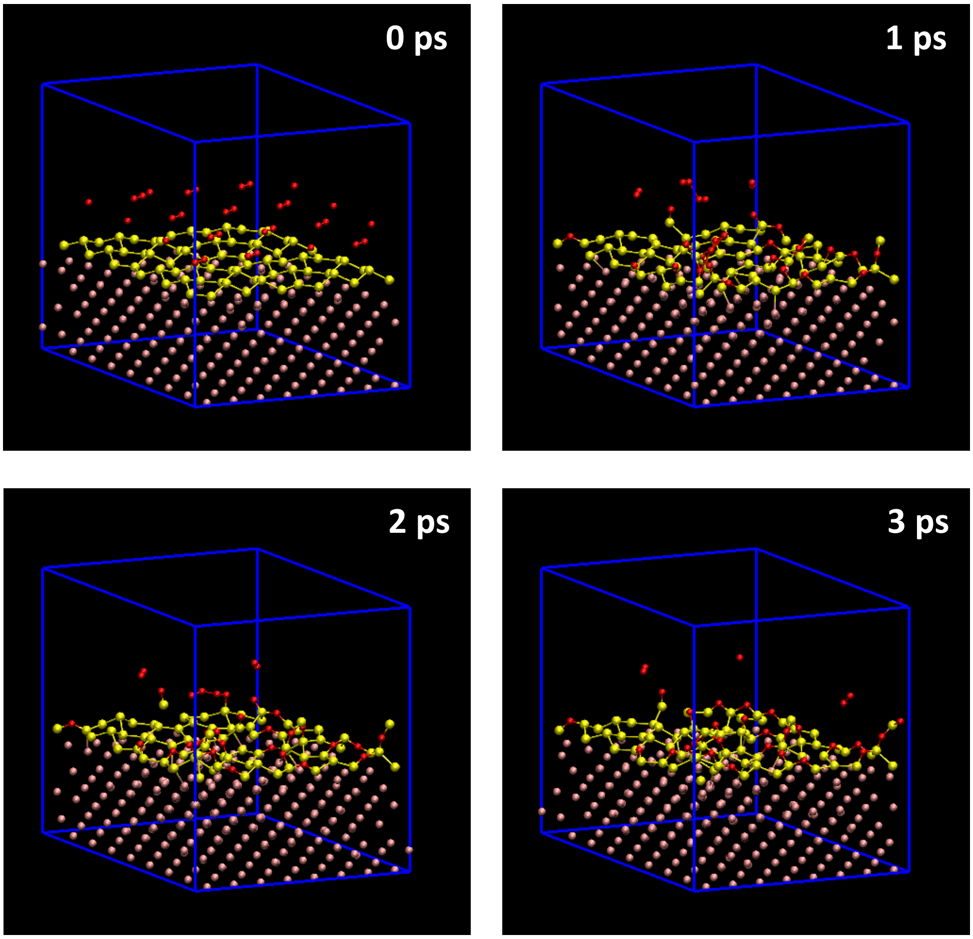

Fig. S1: Snapshots from the FPMD run of oxidation at a high oxygen dose (16 O_2 molecules in the MD box denoted by the blue lines). The red, yellow, and pink atoms indicate oxygen, silicon, and silver atoms, respectively.

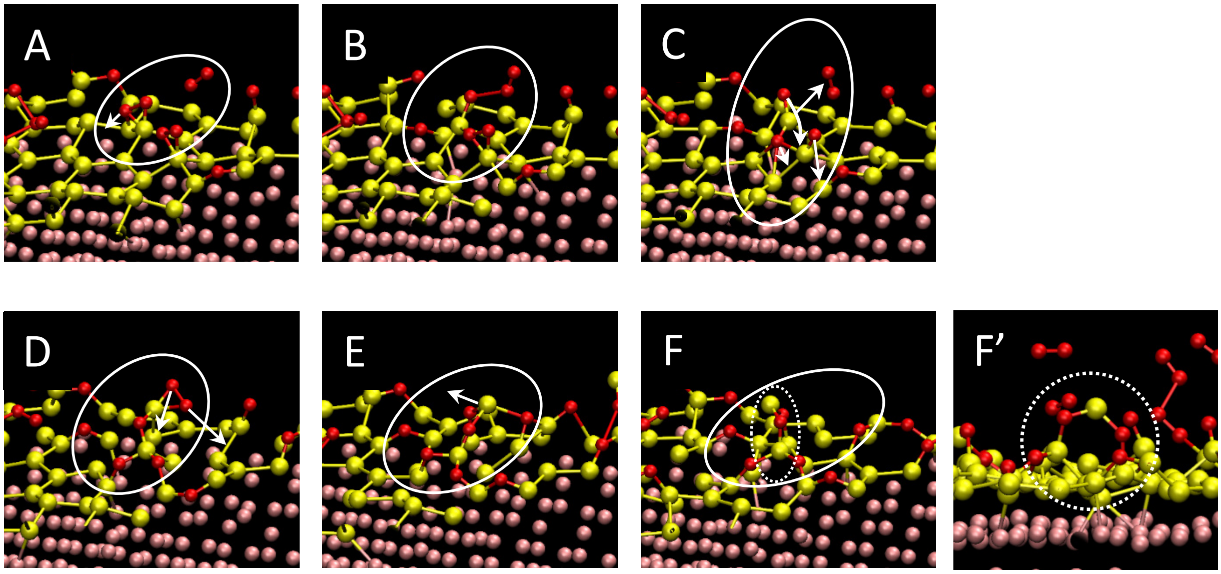

Fig. S2: The sequence of the “chain-like reaction” shown in Fig. 4. Each panel shows the same atomic configurations displayed in the corresponding panels of Fig. 4, but viewed from a different angle. The white circles and arrows denote the same oxygen atoms or their displacements, respectively, as in the panels of Fig. 4 (Panel F’ displays the side view of Panel F).

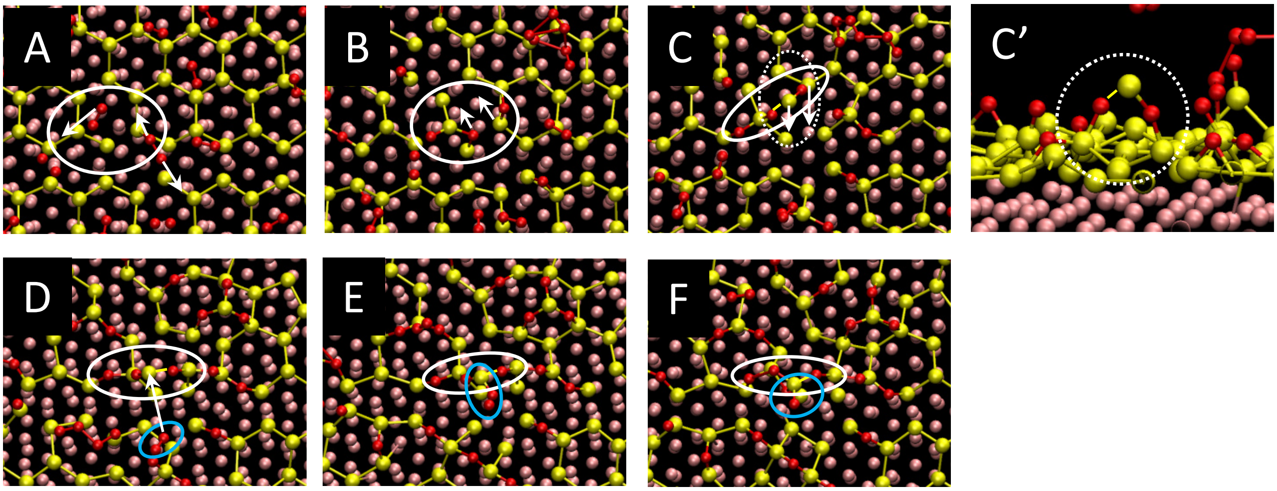

Fig. S3: Another sequence of the “chain-like reaction” observed in our FPMD simulation. In the same way as in Fig. 4 and Fig. S2, the white arrows indicate where an oxygen atom will move to, and the white circle focuses the important process being shown in each panel. Panel C’ displays the side view of Panel C, where a 3D-like structural configuration is indicated by the white dashed circle. The blue ellipse in Panel D, E, F indicates an O2 molecule that approaches the reaction site [D], then bonds to the site [E], and dissociates [F].
